# Projected reduction in major adverse cardiovascular events among high-risk U.S. adults with type 2 diabetes eligible for oral semaglutide: a SOUL trial–based analysis using NHANES (1988–2018) cycles

**DOI:** 10.1186/s40842-026-00299-z

**Published:** 2026-05-22

**Authors:** Mustafa Al-jarshawi, Andrew Cole, Rodrigo Bagur, Hao-Yu Wang, Cheng Han Ng, Dahai Yu, Nicholas W. S. Chew, Mamas A. Mamas

**Affiliations:** 1 Centre for Health Informatics, Division of Informatics, Imaging and Data Science, Faculty of Biology, Medicine and Health, University of Manchester, Manchester, UK; 2 Keele Cardiovascular Research Group, Centre for Prognosis Research, Keele University, Keele, UK; 3 Department of Cardiology, London Health Sciences Centre, Western University, London, ON Canada; 4 Coronary Heart Disease Center and Cardiometabolic Medicine Center, Department of Cardiology, Fuwai Hospital, National Center for Cardiovascular Diseases, Chinese Academy of Medical Sciences and Peking Union Medical College, Beijing, China; 5 Division of Gastroenterology and Hepatology, Department of Medicine, National University Hospital, Singapore, Singapore; 6 School of Medicine, Keele University, Keele, UK; 7 Department of Cardiology, National University Heart Centre, National University Health System, Singapore, Singapore; 8 Honorary Clinical Lecturer, Institute of Health Sciences Education, Queen Mary University of London, London, UK; 9 Council Member, European Society of Cardiology (ESC) Council of Cardio-Oncology, Sophia Antipolis, France; 10 NIHR Academy member, National Institute for Health & Care Research, Leeds, UK

**Keywords:** Oral semaglutide, Type 2 diabetes, Major adverse cardiovascular events, SOUL trial, NHANES, National health and nutrition examination survey, Secondary prevention, Glucagon-like peptide-1 receptor agonist, Population health modelling, Cardiovascular risk reduction

## Abstract

**Background:**

Oral semaglutide, a glucagon-like peptide-1 receptor agonist (GLP-1 RA), has been shown to reduce major adverse cardiovascular events (MACE) in high-risk individuals with type 2 diabetes in the SOUL trial. Its potential population-level impact in the United States, however, remains unclear.

**Methods:**

We applied SOUL trial eligibility criteria to U.S. adults in the National Health and Nutrition Examination Survey (NHANES, 1988–2018) to estimate the number eligible for oral semaglutide and the projected reduction in cardiovascular events over 4.5 years. MACE—defined as a composite of cardiovascular death, nonfatal myocardial infarction, and nonfatal stroke—was the primary outcome. Event rates and hazard ratios were derived from the SOUL trial and applied to weighted NHANES data.

**Results:**

An estimated 6.1 million U.S. adults met eligibility criteria for oral semaglutide. Without treatment, 847,598 MACE events were projected, compared to 728,934 with treatment—corresponding to 118,664 events potentially prevented (14% relative reduction; number needed to treat [NNT] = 55). This included 28,430 cardiovascular deaths (NNT = 254), 83,489 nonfatal myocardial infarctions (NNT = 78), and 24,521 nonfatal strokes (NNT = 284). Cardiovascular benefits were consistent across sex, race/ethnicity, and clinical subgroups. At 50% uptake, and assuming a 15.5% discontinuation rate as observed in the SOUL trial, an estimated 59,332 MACE, 14,215 cardiovascular deaths, 41,744 nonfatal myocardial infarctions, and 12,260 nonfatal strokes could be prevented.

**Conclusions:**

Widespread use of oral semaglutide among eligible U.S. adults with type 2 diabetes could substantially reduce cardiovascular events. These findings support its potential as a scalable, population-level cardiometabolic intervention and highlight the need for system-level strategies to enhance access and uptake.

**Supplementary Information:**

The online version contains supplementary material available at 10.1186/s40842-026-00299-z.

## Background

Diabetes affects an estimated 828 million adults worldwide, with a global age-standardised prevalence of approximately 14% among adults [1]. Obesity, a key modifiable risk factor for type 2 diabetes, has risen steadily over the past few decades in most parts of the world. By 2035, more than half of the global population is projected to be living with overweight or obesity [2]. The health impact is profound: in 2015, obesity was linked to an estimated 4 million deaths globally, with over two-thirds of these deaths due to cardiovascular diseases [2]. At the same time, diabetes was the second leading cause of obesity-related deaths that year, accounting for approximately 0.6 million deaths and 30.4 million disability-adjusted life-years. Notably, 9.5% of these diabetes-related deaths occurred in individuals with a BMI of 30 kg/m^2^ or higher, while 4.5% occurred at a BMI below 30 kg/m^2^.

As a result, therapeutic strategies that simultaneously target glycaemic control and weight reduction have become increasingly important in managing overall cardiometabolic risk. Glucagon-like peptide-1 (GLP-1) receptor agonists are a class of medications used for managing type 2 diabetes and obesity [3]. Semaglutide, a potent GLP-1 receptor agonist, has shown notable results: the SELECT trial demonstrated that weekly subcutaneous semaglutide 2.4 mg significantly reduced the risk of major adverse cardiovascular events (MACE) even in individuals without diabetes but with established cardiovascular disease and overweight or obesity [4].

The cardiovascular safety of oral semaglutide has been established in individuals with type 2 diabetes [5]. The recent Semaglutide Cardiovascular Outcomes Trial (SOUL) reported that once-daily oral semaglutide (maximal dose, 14 mg), in addition to standard care, significantly reduces the risk of MACE in individuals with type 2 diabetes and atherosclerotic cardiovascular disease, chronic kidney disease, or both, without an increase in the incidence of serious adverse events [6]. However, the potential population-level impact of this intervention when scaled to the US population is unknown. In this study, we aim to evaluate the real-world implications of the SOUL trial by estimating (a) the number of U.S. adults who would be eligible for oral semaglutide based on SOUL trial inclusion criteria, and (b) the potential reduction in cardiovascular events across the eligible U.S. population with widespread use of oral semaglutide.

## Methods

### Study design and data sources

We conducted a cross-sectional, population-based modelling study using publicly available data from the U.S. National Health and Nutrition Examination Survey (NHANES) cycles (1988–2018). The National Centre for Health Statistics, part of the CDC, produces this survey aimed at monitoring the health of the US population. The NHANES is a major programme that generates a nationally representative sample of the civilian non-institutionalized US population from about 5000 individuals each year in a 2-year cycle. These are generated through complex, multi-stage, probability sampling process with oversampling of specific subgroups to improve the reliability and accuracy in these populations [7]. Detailed sampling and data collection procedures have been previously published [8]. In addition to demographics, socioeconomic, and health-related questions, the surveys also include medical, physiological, and laboratory measurements.

### Study population

#### Inclusion criteria

Participants were included according to the eligibility criteria defined in the SOUL trial (supplementary Fig. 1). Individuals were eligible for inclusion if they met al.l of the following conditions: [1] aged 50 years or older; [2] diagnosed with type 2 diabetes; [3] had a glycated hemoglobin (HbA1c) level between 6.5% and 10.0%, inclusive; and [4] had at least one of the following comorbid conditions: coronary artery disease (e.g., history of myocardial infarction), cerebrovascular disease (e.g., prior stroke or transient ischemic attack), symptomatic peripheral artery disease, or chronic kidney disease, defined as an estimated glomerular filtration rate (eGFR) less than 60 mL/min/1.73 m².

#### Exclusion criteria

Participants were excluded if they met any of the following criteria, based on self-reported data or clinical/laboratory measures available in NHANES: [1] age under 50 years; [2] diagnosis of type 1 diabetes; [3] history of cancer within the past five years; [4] eGFR < 15 mL/min/1.73 m², indicating end-stage kidney disease; [5] recent major cardiovascular events within the past 60 days, such as myocardial infarction, stroke, or hospitalisation for unstable angina.

### Baseline characteristics

Baseline characteristics were collected through standardised questionnaires and objective measurements conducted in Mobile Examination Center (MEC) assessments. Age was recorded as a continuous variable, and sex was classified as male or female. Race and ethnicity were self-reported and categorised as Mexican American, Other Hispanic, Non-Hispanic White, Non-Hispanic Black, or Other Race (including multiracial individuals). Type 2 diabetes was defined by a self-reported diagnosis or current use of oral hypoglycaemic agents. Hypertension was defined by a self-reported diagnosis or current use of antihypertensive medication. Hyperlipidaemia was identified through a self-reported history of high cholesterol or current use of lipid-lowering therapy. Current smoking was determined based on “Every day” or “Some days” responses to the question, “Do you now smoke cigarettes?”.

Anthropometric and physiological parameters—including weight, height, body mass index (BMI), blood pressure, and pulse rate—were measured during MEC visits following NHANES standard protocols. BMI was calculated as weight in kilograms divided by height in meters squared. Blood pressure was measured in accordance with NHANES protocols, with three consecutive readings obtained and the mean value used for analysis. Pulse rate was recorded at rest. Laboratory data were obtained from fasting blood samples and processed according to NHANES quality assurance standards. Glycated haemoglobin (HbA1c) was used to assess glycaemic control. Renal function was evaluated using serum creatinine, with estimated glomerular filtration rate (eGFR) calculated using the Chronic Kidney Disease Epidemiology Collaboration method (CKD-EPI 2009 equation) [9]. Cardiovascular disease was defined as a self-reported diagnosis of at least one of the following: coronary heart disease, angina, myocardial infarction, stroke, or congestive heart failure. Chronic kidney disease was defined as an eGFR < 60 mL/min/1.73 m².

### Study outcomes

The primary outcome was major adverse cardiovascular events (MACE), defined as a composite of cardiovascular death, nonfatal myocardial infarction (MI), or nonfatal stroke, over a 4.5-year follow-up period, consistent with the SOUL trial. Secondary outcomes were the individual components of MACE.

### Statistical analysis

The statistical analysis framework in this study is consistent with prior work by Lusk et al. [10], which applied trial-derived hazard ratios to NHANES populations to project population-level cardiovascular event reductions in the SELECT trial. We estimated the number of adults in the U.S. population who met the SOUL trial inclusion criteria by applying NHANES sample weights. Combined weights for the 1998–2018 survey cycles were calculated using NHANES-recommended formulae and subsequently rounded to the nearest integer. A detailed explanation of the weighting methodology is available on the NHANES website (https://wwwn.cdc.gov/nchs/nhanes/tutorials/weighting.aspx*).* Continuous variables were reported as medians with interquartile ranges (IQR), and categorical variables as proportions.

We set the national incidence of the primary MACE endpoint over 54 months equal to that observed in the placebo arm of the SOUL trial (668 events among 4,825 participants, or 13.844%). This incidence rate was multiplied by the weighted NHANES population meeting the SOUL trial eligibility criteria to estimate the expected number of MACE events without treatment. To estimate the number of MACE events potentially preventable with oral semaglutide, we applied the hazard ratio (HR) from the SOUL trial (supplementary Table 1) to the incidence rate. As the HR was derived from an intention-to-treat analysis, it reflected real-world patterns of adherence and discontinuation observed in the trial. The same approach was followed for each secondary outcome—cardiovascular death, nonfatal myocardial infarction, and nonfatal stroke—using the corresponding event rates and hazard ratios from the SOUL trial. In the SOUL trial, non-cardiovascular death was treated as a censoring event in the Cox proportional hazards model; accordingly, we applied the hazard ratios from this intention-to-treat analysis to our population estimates without additional competing risk adjustment. We also calculated the number needed to treat (NNT), defined as the number of individuals who would need to receive oral semaglutide over 4.5 years to prevent one event. NNT was computed as the reciprocal of the absolute risk reduction for each outcome.

We conducted subgroup analyses by sex, race/ethnicity, and comorbidity group (CKD only vs. CVD only) to estimate the impact of oral semaglutide across different populations. These analyses assumed a consistent relative treatment effect across subgroups. While the SOUL trial was not powered to detect differences between subgroups, the treatment effect appeared consistent across them.

As a sensitivity analysis, we conducted additional scenario modelling across varying levels of oral semaglutide uptake, assessed in 10% increments from 0% to 100%. For each uptake level, the total number of endpoint events was estimated as the sum of: [1] the number of individuals not receiving oral semaglutide multiplied by the endpoint incidence at 54 months, and [2] the number of individuals receiving oral semaglutide multiplied by the hazard ratio–adjusted incidence. Given that the event rate for the composite MACE outcome was below 15%, the HR was assumed to approximate the relative risk. Therefore, multiplying the HR by the baseline incidence yielded the estimated risk among treated individuals, and the difference in event totals between treated and untreated groups represented the number of events potentially prevented.

We also estimated projected changes in body weight and HbA1c associated with oral semaglutide use by applying mean differences observed in the SOUL trial between baseline and 104 weeks in both treatment and placebo arms, to estimate potential metabolic improvements at the population level.

### Ethical considerations

This study adhered to the ethical principles for medical research as outlined in the Declaration of Helsinki. The NHANES datasets across cycles are publicly available and de-identified. This study was exempt from institutional review board approval. The original survey protocols were approved by the NCHS Research Ethics Review Board, and all participants provided informed consent.

## Results

A total of 6,123,945 U.S. adults are estimated to be eligible to receive once-daily oral semaglutide (maximum dose, 14 mg), based on SOUL trial inclusion criteria applied to NHANES data from 1988 to 2018 - representing approximately 2.4% of the U.S. adult population during the study period.

The median age of the eligible population was 69 years (IQR 63–76), of whom 51% were female. Most individuals were non-Hispanic White (72%), followed by non-Hispanic Black (16%) and Mexican American (4.0%). The cohort had a median BMI of 31 kg/m² (IQR 27–36), and a median HbA1c of 7.5% (IQR 6.9–8.2). Median eGFR was 55 mL/min/1.73 m² (IQR 43–68). CV risk factors were prevalent: 71% had a history of hypertension, 60% had hyperlipidaemia, and 14% reported current smoking. Overall, 60% of eligible individuals had established CVD, including prior myocardial infarction (30%), stroke (20%), coronary heart disease (35%), and heart failure (24%), as outlined in (Table 1). Comparability of baseline characteristics with SOUL trial participants (*N* = 9,650) is summarised in (Supplementary Table 2). The NHANES-eligible population was slightly older (69 vs. 66 years) and included a substantially higher proportion of women (51% vs. 29%) and non-Hispanic Black individuals (16% vs. 2.7%). The prevalence of chronic kidney disease was also higher in NHANES-eligible population (40% vs. 13%), whereas the prevalence of coronary artery disease was considerably lower (35% vs. 71%).

**Table 1:** Baseline characteristics of U.S. adults eligible for oral semaglutide, based on SOUL trial inclusion criteria

Characteristic	*N* = 6,123,945^1^
**Sex**— no. (%)	
Male	2,998,317 (49%)
Female	3,125,628 (51%)
**Age**— yr	69 (63, 76)
**Race/ethnicity**— no. (%)	
Mexican American	246,567 (4.0%)
Other Hispanic	111,593 (1.8%)
Non-Hispanic White	4,437,144 (72%)
Non-Hispanic Black	970,481 (16%)
Other Race - Including Multi-Racial	358,160 (5.8%)
**Weight (kg)**	85 (73, 100)
Body Mass Index (kg/m**2)	31 (27, 36)
**Systolic Blood Pressure (mmHg)**	135 (122, 151)
**Diastolic Blood Pressure (mmHg)**	67 (59, 75)
**Pulse rate (bpm)**	74 (64, 82)
**Glycohemoglobin (%)**	7.50 (6.90, 8.20)
**Estimated Glomerular Filtration Rate (mL/min/1.73 m2)**	55 (43, 68)
**Hypertension**— no. (%)	4,377,401 (71%)
**Current Smoking**— no. (%)	279,867 (14%)
**Hyperlipidaemia**— no. (%)	3,346,014 (60%)
**History of Cardiovascular Disease Only**— no. (%)	3,694,779 (60%)
**History of Kidney Disease Only**— no. (%)	2,429,166 (40%)
**Heart attack**— no. (%)	1,842,112 (30%)
**Stroke**— no. (%)	1,241,420 (20%)
**Coronary Heart Disease**— no. (%)	1,316,951 (35%)
**Angina**— no. (%)	828,934 (22%)
**Heart Failure**— no. (%)	1,490,611 (24%)

^1^Median (Q1, Q3); n (%)

Over a 4.5-year period, an estimated 847,598 MACE would occur in the absence of treatment with oral semaglutide, compared with 728,934 events with treatment—corresponding to 118,664 events potentially prevented (14%), with a number needed to treat (NNT) of 55 (Table 2). For cardiovascular death, the projected number of events was 436,398 without treatment versus 407,968 with treatment, yielding 28,430 deaths potentially prevented (6.5%) (NNT = 254). The estimated reduction in nonfatal myocardial infarctions (MI) was from 345,104 to 261,615 events, resulting in 83,489 potentially preventable events (24%) (NNT = 78). Similarly, the number of nonfatal strokes was projected to decline from 219,290 to 194,769 events, equating to 24,521 potentially preventable strokes (11%) (NNT = 284) with population-wide use of oral semaglutide. These projections were derived by applying incidence rates from the placebo group and HRs reported in the SOUL trial (Supplementary Table 1).

**Table 2:** Projected impact of oral semaglutide on cardiovascular outcomes over 4.5 years among eligible U.S. adults (NHANES 1988–2018), based on the SOUL trial

Outcome	Estimated Events Without Oral Semaglutide	Estimated Events with Oral Semaglutide	Potential Events Prevented	NNT
MACE	847,598	728,934	118,664	55
Cardiovascular death	436,398	407,968	28,430	254
Nonfatal MI	345,104	261,615	83,489	78
Nonfatal Stroke	219,290	194,769	24,521	284

Estimates are based on 6,123,945 U.S. adults identified in NHANES 1988–2018 as eligible for oral semaglutide according to SOUL trial inclusion criteria. Projected events over 4.5 years were calculated by applying incidence rates observed in the placebo group of the SOUL trial (supplementary Table 1) and corresponding hazard ratios: 0.86 for MACE, 0.93 for cardiovascular death, 0.77 for nonfatal myocardial infarction, and 0.89 for nonfatal stroke (see Supplementary Table 1). All figures incorporate NHANES survey weights to provide nationally representative estimates. NNT = number needed to treat over 4.5 years

**Fig. 1:**
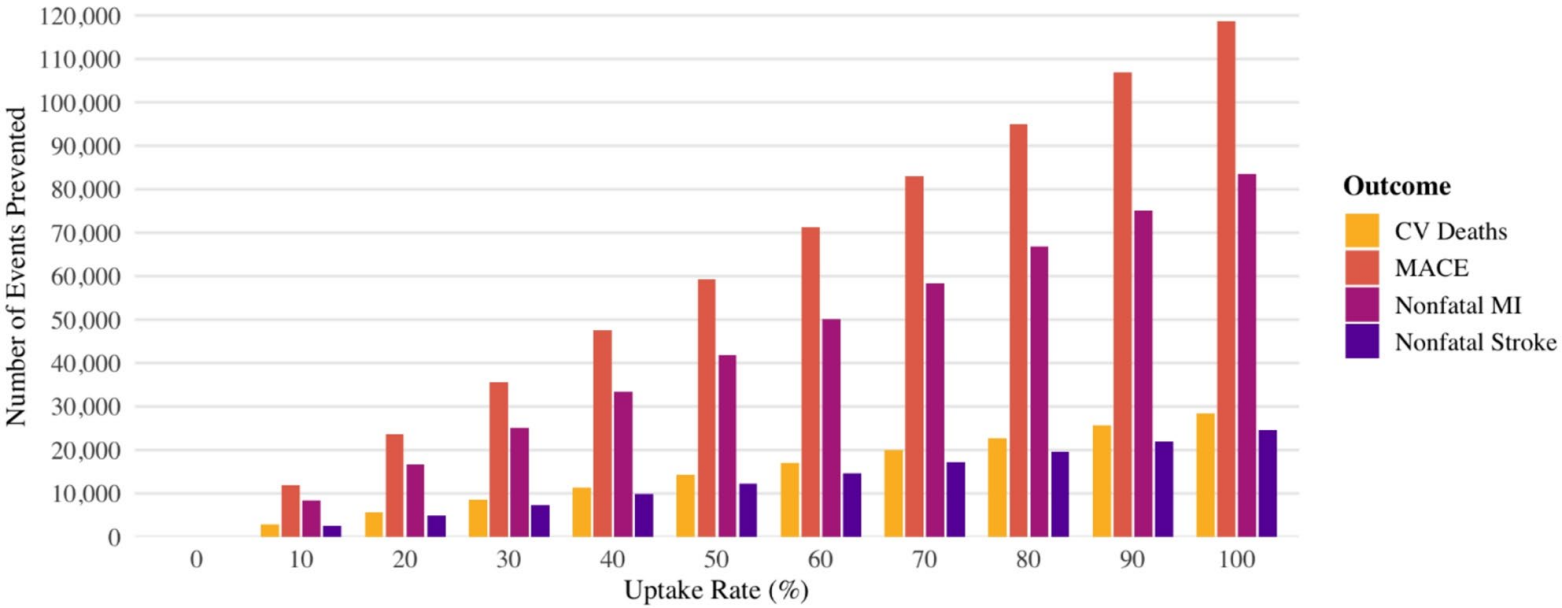
Potential events prevented (4.5-Year Projection) with increasing oral semaglutide uptake among eligible U.S. adults as per SOUL trial in NHANES cycles (1988–2018)

Figure 1 shows the projected number of cardiovascular events that could potentially be prevented over 4.5 years with varying uptake levels of oral semaglutide among the eligible U.S. population identified in NHANES (1988–2018). At 10% uptake, an estimated 11,866 MACE, 2,843 cardiovascular deaths, 8,349 nonfatal myocardial infarctions (MIs), and 2,452 nonfatal strokes could potentially be prevented. At 50% uptake, assuming a discontinuation rate of 15.5% as observed in the SOUL trial, the corresponding estimates were 59,332 MACE, 14,215 cardiovascular deaths, 41,744 nonfatal MIs, and 12,260 nonfatal strokes.

**Fig. 2:**
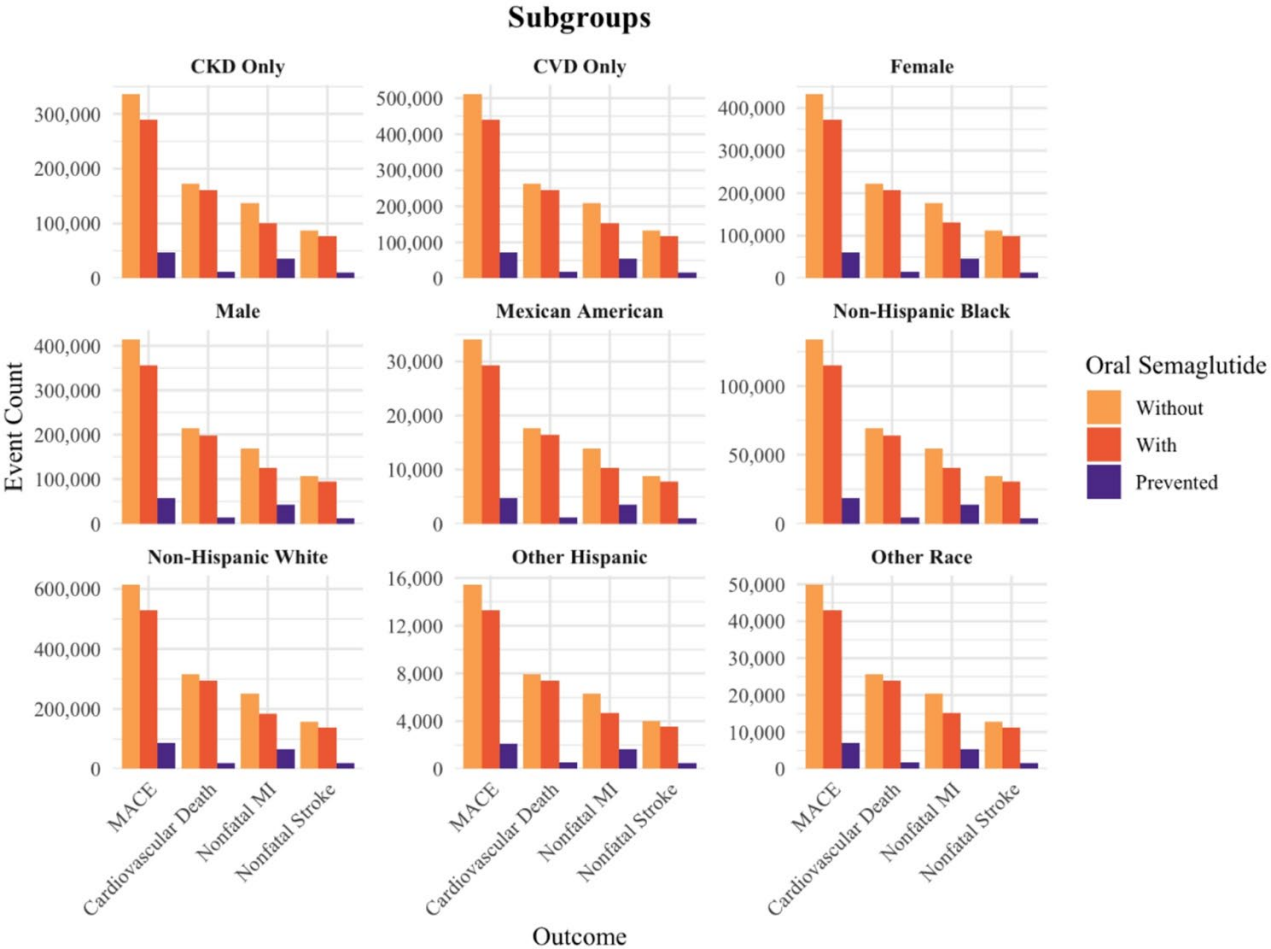
Projected impact of oral semaglutide on cardiovascular outcomes over 4.5 Years among eligible U.S. Adult subgroups (NHANES 1988–2018), based on the SOUL trial

Subgroup analyses showed consistent cardiovascular benefits of oral semaglutide across sex, racial/ethnic, and clinical groups (Fig. 2). Among males (*n* = 2,998,317), treatment was associated with 58,098 fewer MACE (~ 15% relative reduction), 14,956 fewer cardiovascular deaths (~ 7%), 43,931 fewer nonfatal myocardial infarctions (~ 25%), and 12,884 fewer nonfatal strokes (~ 12%). Among females (*n* = 3,125,628), estimated potential reductions were similar: 60,565 MACE (~ 15%), 15,591 cardiovascular deaths (~ 7%), 45,796 nonfatal myocardial infarctions (~ 25%), and 13,431 nonfatal strokes (~ 12%).

While the largest potential absolute benefit by race/ethnicity was observed in non-Hispanic White individuals, with 86,010 MACE (~ 14%), 21,123 cardiovascular deaths (~ 6%), 64,602 nonfatal myocardial infarctions (~ 25%), and 18,864 nonfatal strokes (~ 11%) prevented. The largest potential relative risk reductions were observed in vulnerable minority population groups. Among non-Hispanic Black individuals, estimated reductions included 18,783 MACE (~ 16), 4,840 cardiovascular deaths (~ 7%), 14,116 nonfatal myocardial infarctions (~ 26%), and 4,116 nonfatal strokes (~ 12%). For Mexican American adults, 4,779 MACE (~ 16%), 1,221 cardiovascular deaths (~ 7%), 3,607 nonfatal myocardial infarctions (~ 26%), and 1,058 nonfatal strokes (~ 12%) were prevented. Other Hispanic individuals had 2,157 fewer MACE (~ 14%), 555 fewer cardiovascular deaths (~ 6%), 1,629 fewer nonfatal myocardial infarctions (~ 25%), and 475 fewer nonfatal strokes (~ 11%). Among individuals of other or multi-racial backgrounds, 6,983 MACE (~ 15%), 1,803 cardiovascular deaths, 5,252 nonfatal myocardial infarctions (~ 7%), and 1,532 (~ 26%), nonfatal strokes (~ 12%) were prevented. In those with chronic kidney disease only (*n* = 2,429,166), treatment was projected to prevent 47,081 MACE (~ 15%), 12,115 cardiovascular deaths (~ 6%), 35,583 nonfatal myocardial infarctions (~ 25%), and 10,436 nonfatal strokes (~ 11%). Among individuals with cardiovascular disease only (*n* = 3,694,779), estimated reductions included 71,611 MACE (~ 14%), 18,428 cardiovascular deaths (~ 7%), 54,123 nonfatal myocardial infarctions (~ 25%), and 15,873 nonfatal strokes (~ 12%).

Supplementary Table 3 shows the projected number of cardiovascular events that could have been prevented across NHANES cycles. Over the last two decades, the number of U.S. adults eligible for oral semaglutide increased steadily, rising from 203,384 in the 1999–2000 cycle to 669,469 in the 2017–2018 cycle. This growth in the eligible population was accompanied by a corresponding increase in the number of potentially preventable events. For example, the number of MACE events that could be prevented increased from 3,938 in 1999–2000 to 12,976 in 2017–2018. Over the same period, projected cardiovascular deaths rose from 943 to 3,109, nonfatal myocardial infarctions from 2,812 to 9,282, and nonfatal strokes from 827 to 2,733.

**Fig. 3:**
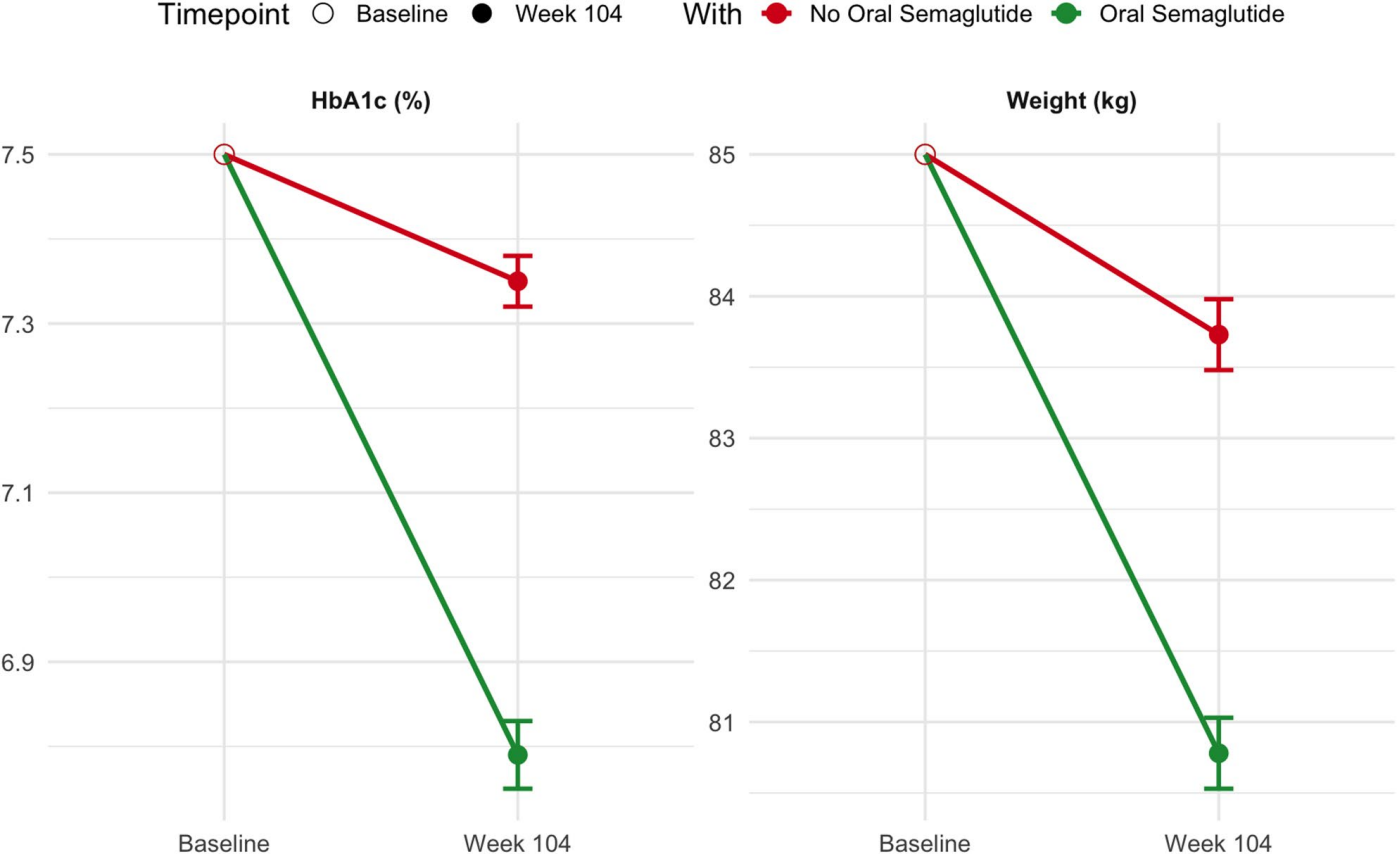
Projected reductions in HbA1c and body weight among eligible U.S. adults (NHANES 1988–2018)*.* n the SOUL trial, the mean change between baseline to 104 weeks with oral semaglutide (14 mg daily) was − 0.71% for HbA1c (95% CI: − 0.75 to − 0.67) and − 4.22 kg for body weight (95% CI: − 4.47 to − 3.97), compared to − 0.15% (95% CI: − 0.18 to − 0.12) and − 1.27 kg (95% CI: − 1.52 to − 1.02), respectively, in the placebo group. Confidence intervals for the NHANES projections incorporate NHANES weighting variance

Finally, among all U.S. adults eligible for this trial, mean HbA1c was projected to decline from 7.5% at baseline to 6.79% at week 104, and body weight from 85.0 kg to 80.8 kg with the use of oral semaglutide, based on treatment effects observed in the SOUL trial (Fig. 3).

## Discussion

In this nationally representative modelling study, an estimated 6.1 million U.S. adults (approximately 2.4% of the adult population) met eligibility criteria for oral semaglutide based on the SOUL trial. Over 27.6 million person-years of follow-up, full uptake of oral semaglutide was projected to prevent approximately 118,664 major adverse cardiovascular events (MACE)—equivalent to 430 prevented events per 100,000 person-years. This included 28,430 cardiovascular deaths (103 per 100,000 person-years), 83,489 nonfatal myocardial infarctions (303 per 100,000 person-years), and 24,521 nonfatal strokes (89 per 100,000 person-years), with a number needed to treat (NNT) of 55 for MACE. Even at 50% uptake, substantial benefits were observed, with over 59,000 MACE and 14,000 cardiovascular deaths potentially prevented.

Importantly, although our primary estimates are derived from pooled NHANES data spanning 1988–2018, we also examined the most recent cycle (2017–2018) to provide contemporary context. In this cycle, approximately 669,469 U.S. adults were eligible for oral semaglutide, among whom an estimated 12,976 major adverse cardiovascular events (MACE)—including 3,109 cardiovascular deaths, 9,282 nonfatal myocardial infarctions, and 2,733 nonfatal strokes—could potentially be prevented over 4.5 years. These findings highlight that the projected cardiovascular benefits remain substantial in a contemporary population. However, increasing use of cardioprotective therapies (e.g., statins, SGLT2 inhibitors) and improved risk factor control over time may lower baseline event rates, potentially attenuating the absolute number of preventable events. Accordingly, our pooled estimates may represent an upper-bound projection of population-level benefit.

Compared to the SOUL trial population, the U.S. population eligible for oral semaglutide in NHANES included a higher proportion of women and non-Hispanic Black individuals, as well as a greater burden of chronic kidney disease and a lower prevalence of coronary artery disease (CKD: 40% vs. 13%; CAD: 35% vs. 71%). However, the SOUL trial reported broadly consistent treatment effects for the primary MACE outcome across prespecified subgroups, including CKD-only, CAD-only, and combined CKD/CVD groups (Fig. S4, SOUL trial appendix), supporting the applicability of the relative treatment effect across these high-risk populations. Nevertheless, differences in baseline clinical characteristics between trial participants and real-world populations may still influence generalisability, particularly as the trial was not powered to detect subgroup differences. Among those eligible, we also observed larger potential relative risk reductions among non-Hispanic Black and Mexican American individuals. These differences suggest that broader implementation of oral semaglutide in real-world practice could potentially yield even greater public health benefits, by addressing health disparities among groups historically underrepresented in clinical trials.

It remains plausible that the reductions in MACE observed with oral semaglutide in the SOUL trial—and as projected in our population-level modelling—are at least partly attributable to direct GLP-1 receptor signalling, given the receptor’s expression in cardiovascular tissues and its downstream effects [11]. Beyond glycaemic control, semaglutide reduces body weight by delaying gastric emptying and promoting satiety, lowers blood pressure, improves lipid profiles, and dampens systemic inflammation—all of which are critical contributors to atherosclerotic cardiovascular disease (ASCVD) [12]. Preclinical studies also support direct vascular and myocardial benefits, including enhanced endothelial function, reduced oxidative stress, and improved cardiac metabolism [13, 14].

Although subcutaneous semaglutide is already approved by the FDA for cardiovascular risk reduction in adults with type 2 diabetes and established atherosclerotic cardiovascular disease, oral semaglutide is currently approved only for glycaemic control. While PIONEER 6 demonstrated cardiovascular safety (non-inferiority), and the SOUL trial reported significant reductions in MACE, the oral formulation does not yet carry a formal FDA indication for cardiovascular risk reduction in high-risk adults with type 2 diabetes. Our findings suggest that, if formally approved for cardiovascular risk reduction, oral semaglutide could meaningfully expand access to early, effective cardiometabolic therapy—particularly for patients who may prefer oral agents over injectable formulations.

A recent systematic review and meta-analysis concluded that semaglutide is generally cost-effective compared with other glucose-lowering therapies, particularly in high-income countries and when assessed over a lifetime horizon [15]. Moreover, the findings were sensitive to factors such as HbA1c reduction, treatment cost, discount rate, and time horizon, and did not differentiate between oral and injectable formulations. In contrast, a recent multicentre real-world study from Italy comparing matched cohorts of oral and injectable semaglutide users reported that oral semaglutide incurred an annual treatment cost approximately 9.3% higher than the injectable version (approximately €1615 vs. €1477 per patient per year), with comparable glycaemic and weight outcomes over 18 months [16]. These cost differences highlight the need for direct head-to-head cost-effectiveness analyses to inform value-based prescribing between the two formulations.

In our analysis, applying SOUL trial treatment effects to eligible U.S. adults projected a decline in mean HbA1c from 7.5% to 6.79% and body weight from 85.0 kg to 80.8 kg over 104 weeks. Without treatment, reductions were more modest (HbA1c: 7.35%; weight: 83.7 kg). These findings are in line with the metabolic benefits observed across major semaglutide trials. In the SOUL trial itself, oral semaglutide (14 mg daily) reduced HbA1c by − 0.71% (95% CI: − 0.75 to − 0.67) and body weight by − 4.22 kg (95% CI: − 4.47 to − 3.97), compared with − 0.15% and − 1.27 kg with placebo. In PIONEER-6, similar improvements were observed over 15.9 months: HbA1c and weight declined by − 1.0% and − 4.2 kg, respectively, versus − 0.3% and − 0.8 kg with placebo. Likewise, SUSTAIN-6 demonstrated that once-weekly subcutaneous semaglutide (0.5 or 1.0 mg) achieved HbA1c reductions of 1.0%–1.4% and weight loss of 3.6–4.5 kg over 2.1 years, compared with smaller reductions in the placebo group.

Our findings carry three key public health implications. First, they highlight the substantial population-level benefit of oral semaglutide in reducing MACE and cardiovascular health disparities among high-risk adults with type 2 diabetes, supporting its potential as a scalable cardiometabolic intervention. Second, the oral formulation provides an alternative to injectable GLP-1 receptor agonists, potentially improving adherence and enabling earlier initiation of cardiovascular risk-reducing therapy in routine practice. Third, with a growing eligible population and an increasing burden of preventable events, system-level strategies—such as coverage expansion, clinician education, and targeted implementation—are essential to realise the full clinical and economic value of oral semaglutide. Our analysis showed that the number of cardiovascular events prevented increased linearly with higher oral semaglutide uptake, reflecting the proportional relationship between treatment coverage and population-level benefit.

### Strengths and limitations

This study has several strengths. First, it is the first to apply SOUL trial eligibility criteria to a nationally representative U.S. dataset (NHANES), allowing for population-level estimates of both treatment eligibility and the projected cardiovascular and metabolic benefits of oral semaglutide. Second, we used effect sizes from a large, contemporary cardiovascular outcomes trial and conducted subgroup analyses to explore potential differences in benefit across demographic and clinical groups. Third, our modelling incorporated projected uptake scenarios, enhancing the practical relevance of our findings. Finally, the use of NHANES data spanning three decades (1988–2018) enabled a longitudinal view of trends in treatment eligibility and benefit. This study also has limitations. While we applied SOUL trial criteria to NHANES data, some key clinical variables were either missing or inconsistently collected. For example, data on peripheral artery disease (PAD) were unavailable, possibly underestimating eligibility. Consequently, the estimated 6.1 million eligible U.S. adults—and the corresponding projected number of preventable cardiovascular events—may represent conservative estimates. However, prior evidence suggests that one-third of individuals with PAD also have other forms of CVD, which may have partially mitigated this exclusion [17]. Second, our subgroup projections assumed uniform treatment effects, although real-world responses may vary by patient characteristics. Third, our projections did not account for the time-varying uptake and intensification of other cardioprotective therapies, including statins, ACE inhibitors, and SGLT2 inhibitors, which have become increasingly common in routine clinical practice and are known to reduce cardiovascular risk in high-risk adults with type 2 diabetes. As NHANES does not provide longitudinal or detailed medication data across cycles, we were unable to incorporate these dynamic background treatment patterns into our analysis. Greater background use of these therapies in contemporary practice may reduce baseline event rates and attenuate the absolute number of preventable events. Fourth, our modelling applied a uniform baseline incidence rate derived from the placebo arm of the SOUL trial to the NHANES population to establish the baseline risk. However, the NHANES-eligible population differs from the trial cohort in key clinical characteristics, including a higher prevalence of chronic kidney disease and a lower prevalence of coronary artery disease. These differences may result in variation in underlying absolute cardiovascular risk in real-world settings compared with the trial population. As a result, the absolute risk reductions and corresponding number needed to treat (NNT) estimated in our analysis may be over- or under-estimated, depending on the true baseline risk distribution. Additionally, our analysis did not account for the competing risk of non-cardiovascular death. Given the relatively older population (median age 69 years), non-cardiovascular mortality over the 4.5-year follow-up period may reduce the number of individuals at risk for cardiovascular events, potentially leading to modest overestimation of projected event rates. Fifth, our projections did not include kidney-specific endpoints, such as the composite of major kidney disease events reported in the SOUL trial, as these were outside the scope of our analysis. However, this may overlook additional potential renal benefits of oral semaglutide at the population level, which should be explored in future research. Sixth, eGFR was calculated using the CKD-EPI 2009 equation to align with the SOUL trial. However, this equation includes a race coefficient that may overestimate eGFR in non-Hispanic Black individuals and underestimate CKD prevalence compared with the newer CKD-EPI 2021 equation, which removes race adjustment and is recommended by current KDIGO guidelines. Consequently, the number of individuals classified as having CKD—and therefore eligible for oral semaglutide—may differ if the CKD-EPI 2021 equation were applied. Finally, while our projections were based on SOUL trial effect sizes, real-world differences in adherence, comorbidities, and access to care may limit generalisability.

## Conclusion

Widespread use of oral semaglutide among eligible U.S. adults with type 2 diabetes could substantially reduce cardiovascular events. These findings support its potential as a scalable, population-level cardiometabolic intervention and highlight the need for system-level strategies to enhance access and uptake.

## Supplementary Information

Below is the link to the electronic supplementary material.

Supplementary Material 1

## Data Availability

The data underlying this article are available in NAHNES website, at [ https://wwwn.cdc.gov/nchs/nhanes/].

## Abbreviations

ASCVD: Atherosclerotic cardiovascular disease
BMI: Body mass index
BRC: Biomedical Research Centre
CAD: Coronary artery disease
CDC: Centers for Disease Control and Prevention
CI: Confidence interval
CKD: Chronic kidney disease
CKD-EPI: Chronic Kidney Disease Epidemiology Collaboration
CVD: Cardiovascular disease
eGFR: Estimated glomerular filtration rate
FDA: Food and Drug Administration
GLP-1: Glucagon-like peptide-1
GLP-1 RA: Glucagon-like peptide-1 receptor agonist
HbA1c: Glycated haemoglobin
HR: Hazard ratio
IQR: Interquartile range
KDIGO: Kidney disease: improving global outcomes
MACE: Major adverse cardiovascular events
MEC: Mobile Examination Center
MI: Myocardial infarction
NCHS: National Center for Health Statistics
NHANES: National Health and Nutrition Examination Survey
NIHR: National Institute for Health and Care Research
NNT: Number needed to treat
PAD: Peripheral artery disease
SOUL: Semaglutide Cardiovascular Outcomes Trial
U.S.: United States
UK: United Kingdom
