# Supplementary material for: Projected reduction in major adverse cardiovascular events among high-risk U.S. adults with type 2 diabetes eligible for oral semaglutide: a SOUL trial–based analysis using NHANES (1988–2018) cycles
Source: Cardiovasc Diabetol Endocrinol Rep. 2026 May 22;12:33. doi: 10.1186/s40842-026-00299-z (PMC13196176; doi:10.1186/s40842-026-00299-z)
Supplement: Supplementary file 1 — Supplementary Material 1 [file 40842_2026_299_MOESM1_ESM.docx]

**Supplementary table 1: Hazard ratios (HRs), 95% confidence intervals (CIs), and p-values for primary endpoints reported in the SOUL trial (N= 4825 per arm: Oral Semaglutide vs Placebo)**

| **Outcome** | **Hazard Ratio (HR)** | **95% Confidence Interval (CI)** | **p-value** | **Events**  **(Oral Semaglutide/Placebo)** |
| --- | --- | --- | --- | --- |
| **MACE** | 0.86 | 0.77–0.96 | 0.006 | 579/668 |
| **Cardiovascular death** | 0.93 | 0.80–1.09 | **—** | 301/ 320 |
| **Nonfatal MI** | 0.74 | 0.61–0.89 | **—** | 191/ 253 |
| **Nonfatal stroke** | 0.88 | 0.70–1.11 | **—** | 144/ 161 |

| **Supplementary table 2: Baseline Characteristics of the SOUL Trial Oral Semaglutide and Placebo Groups Compared with the NHANES-Eligible U.S. Population** | | | |
| --- | --- | --- | --- |
| **Characteristic** | **SOUL Oral Semaglutide Group (N = 4825)** | **SOUL Placebo Group**  **(N = 4825)** | **NHANES Eligible Population**  **N = 6,123,945** |
| **Age — yr** | 66.1±7.6 | 66.1±7.5 | 69 ± 9 |
| **Female sex — no. (%)** | 1376 (28.5) | 1414 (29.3) | 3,125,628 (51%) |
| **Race or ethnic group — no. (%)** |  |  |  |
| White | 3327 (69.0) | 3321 (68.8) | 4,437,144 (72%) |
| Black | 124 (2.6) | 128 (2.7) | 970,481 (16%) |
| Asian | 1134 (23.5) | 1121 (23.2) | - |
| American Indian or Alaska Native | 7 (0.1) | 12 (0.2) | - |
| Native Hawaiian or Pacific Islander | 4 (<0.1) | 5 (0.1) | - |
| Other | 185 (3.8) | 192 (4.0) | 358,160 (5.8%) |
| Not reported | 44 (0.9) | 46 (1.0) | - |
| Hispanic or Latino ethnic group — no. (%)† | 674 (14.0) | 706 (14.6) | 358,160 (5.8%) |
| **Body weight — kg** | 87.5±19.1 | 88.3±19.6 | 88 ± 22 |
| **Body-mass index‡** | 31.0±5.7 | 31.2±5.9 | 32 ± 7 |
| **Glycated hemoglobin level — %** | 8.0±1.2 | 8.0±1.1 | 7.66 ± 0.91 |
| **Cardiovascular disease only§** | 2730 (56.6) | 2738 (56.7) | 3,694,779 (60%) |
| **Chronic kidney disease only§** | 632 (13.1) | 609 (12.6) | 2429166 (40%) |
| **Hypertension — no. (%)** | 4378 (90.7) | 4381 (90.8) | 4,377,401 (71%) |
| **Current smoking — no. (%)** | 545 (11.3) | 584 (12.1) | 279,867 (14%) |
| **Systolic blood pressure — mm Hg** | 134.6±16.3 | 134.7±16.4 | 138 ± 22 |
| **Diastolic blood pressure — mm Hg** | 76.6±10.1 | 76.7±10.1 | 67 ± 14 |
| **Pulse — beats/min** | 72.8±11.1 | 72.9±11.4 | 74 ± 13 |
| **eGFR — ml/min/1.73 m²** | 74.0 ± 22.6 | 73.6 ± 22.4 | 58 ± 20 |
| **Coronary artery disease— %** | 3406 (70.6) | 3415 (70.8) | 1,316,951 (35%) |
| **Heart attack— %** | 1944 (40.3) | 1917 (39.7) | 1,842,112 (30%) |
| **Stroke— %** | 743 (15.4) | 745 (15.4) | 1,241,420 (20%) |
| **Heart failure— %** | 1105 (22.9) | 1124 (23.3) | 1,490,611 (24%) |

* Plus–minus values are means ±SD. Percentages may not total 100 because of rounding. The abbreviation eGFR denotes estimated glomerular filtration rate. † Race and ethnic group were reported by the participant. ‡ The body-mass index is the weight in kilograms divided by the square of the height in meters. § For 3.3% of the participants, whether only one criterion or two criteria were fulfilled was unknown in SOUL Trial. Chronic kidney disease was defined by an eGFR of less than 60 ml per minute per 1.73 m2 ; the most recent eGFR available in the medical record was used in SOUL trial if it had been obtained within the previous 6 months. ¶ The eGFR was measured in SOUL Trial at randomisation with the use of the Chronic Kidney Disease Epidemiology Collaboration method.

**Supplementary table 3: Potential Events Prevented in U.S. eligible adults by NHANES Cycle**

| **NHANES Cycle** | **Eligible U.S. Adults** | **MACE** | **CV Death** | **Nonfatal MI** | **Nonfatal Stroke** |
| --- | --- | --- | --- | --- | --- |
| NHANES III | 2,314,857 | 44,832 | 10,740 | 32,005 | 9,412 |
| 1999–2000 | 203,384 | 3,938 | 943 | 2,812 | 827 |
| 2001–2002 | 210,900 | 4,087 | 980 | 2,916 | 858 |
| 2003–2004 | 342,564 | 6,638 | 1,591 | 4,742 | 1,395 |
| 2005–2006 | 286,999 | 5,562 | 1,334 | 3,975 | 1,168 |
| 2007–2008 | 367,179 | 7,121 | 1,707 | 5,088 | 1,495 |
| 2009–2010 | 342,776 | 6,640 | 1,592 | 4,745 | 1,397 |
| 2011–2012 | 401,896 | 7,786 | 1,866 | 5,571 | 1,641 |
| 2013–2014 | 491,282 | 9,523 | 2,282 | 6,802 | 2,003 |
| 2015–2016 | 492,638 | 9,549 | 2,288 | 6,822 | 2,009 |
| 2017–2018 | 669,469 | 12,976 | 3,109 | 9,282 | 2,733 |

**Supplementary figure 1: Study flowchart**

**
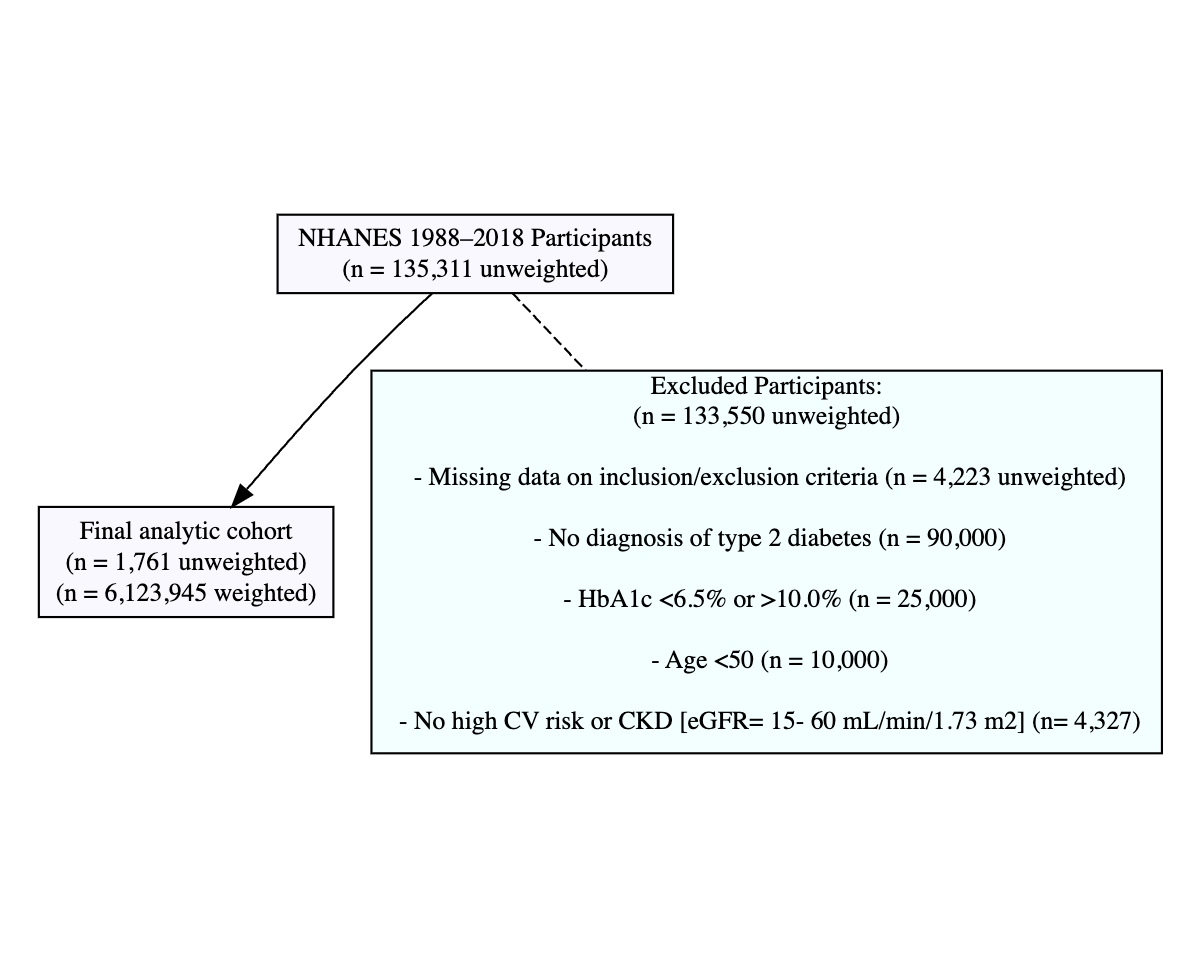
**
